# Safety and Efficacy of Combined Injection of Pure-μ-Opioid Agonist with Tramadol as an Opioid Induction Agent for Opioid-Naïve Cancer Patients

**DOI:** 10.1089/pmr.2023.0061

**Published:** 2024-08-05

**Authors:** Tetsumi Sato, Shigeki Ono, Tetsu Sato, Rei Tanaka, Yoshiko Kamo, Tomomi Suzuki

**Affiliations:** ^1^ Division of Palliative Medicine, Shizuoka Cancer Center, Shizuoka, Japan.; ^2^ Palliative Care Team, Shizuoka Cancer Center, Shizuoka, Japan.; ^3^ Department of Pharmacy, Shizuoka Cancer Center, Shizuoka, Japan.; ^4^ Faculty of Pharmaceutical Sciences, Shonan University of Medical Sciences, Yokohama, Japan.; ^5^ Patient and Family Support Center, Shizuoka Cancer Center, Shizuoka, Japan.

**Keywords:** cancer pain, μ-opioid receptor agonist, opioid combination therapy, opioid naïve, tramadol

## Abstract

**Background:**

Tramadol is known to provide synergistic analgesia when used in combination with morphine.

**Objectives:**

The aims of this study were: (1) to introduce an opioid combination therapy using pure-μ-opioid receptor agonist (OPI) + tramadol injections (OPI + tramadol) and (2) to elucidate safety and efficacy of this combination therapy for opioid-naïve cancer pain patients.

**Methods:**

Opioid-naïve patients referred to our palliative care team (in Japan) who were unable to take oral medications and received OPI + tramadol as opioid induction agents were retrospectively investigated on the electric medical chart. OPI + tramadol dosage was adjusted to achieve the patient’s pain as Numerical Rating Scale ≤4/10 or Support Team Assessment Schedule-Japanese ≤1. Patients’ demography, doses of OPI and tramadol administered, and adverse events were analyzed.

**Results:**

A total of 44 patients were included. The primary organs of malignancy were pancreas (11), stomach (5), lung (4), breast (4), liver (4), and others (13). OPI injections administered were hydromorphone (39), morphine (6), oxycodone (1), and fentanyl (1). The starting doses of OPI (morphine equivalent) and tramadol were 6.05 ± 1.63 and 67.8 ± 13.6 mg/day, respectively, and the final doses of OPI (morphine equivalent) and tramadol were 8.14 ± 3.85 and 80.0 ± 28.5 mg/day, respectively. Treatment goals were achieved in all patients. There were three patients in whom OPI was switched owing to inadequate analgesia and no new side effects other than those known to occur when OPI or tramadol is administered appeared.

**Conclusion:**

The results suggest that this innovative and unique opioid therapy can be safely and effectively introduced to opioid-naïve cancer patients who are relatively close to the end of life.

## Introduction

Pain is the most fearful symptom for cancer patients and its incidence is still high.^1,2^ For moderate-to-severe pain, the strong μ-opioid receptor agonist (OPI) is the mainstay of pharmacotherapy,^3,4^ but side effects on the gastrointestinal and central nervous systems may impair the quality of life (QOL) of the patients, often resulting in unsatisfactory pain relief owing to the inability to receive sufficient doses.

Tramadol has inhibitory effects on serotonin and noradrenaline reuptake in the dorsal horn of the spinal cord and is also known as a dual-action analgesic (DAA). This inhibitory effect results in suppression on transmission of nociceptive stimulation.^5–12^ In addition, it has been reported that morphine and tramadol synergistically suppress pain in animal nociceptive pain models,^13,14^ and this combination was reported to reduce morphine dosage in postoperative pain after abdominal hysterectomy.^15^ However, tramadol has only been simply used as a step 2 opioid for cancer pain, and no previous studies have been conducted on the effects and safety of its use in combination with step 3 opioids in palliative setting. Furthermore, for patients who are unable to intake oral medications, few drugs are available as an adjuvant with monoamine reuptake inhibitory effects. Tramadol is available not only as an oral medication but also as an injectable drug.

Since 2016, the authors have applied OPI and DAA combination therapy to the treatment of cancer pain in our hospital, which is a hub medical institution for cancer patients in eastern Shizuoka and adjacent prefectures. For patients who need an opioid analgesic and are unable to take oral medications, we have administered combined OPI and tramadol injections as the first-line therapy to treat their cancer pain. The aims of this retrospective clinical analysis were: (1) to introduce this innovative and unique opioid combination therapy and (2) to investigate its safety and efficacy as opioid induction agents to treat pain in opioid-naïve cancer patients, as the first step to show the usefulness of this combination therapy.

## Methods

### Ethical considerations

This study was approved by the Shizuoka Cancer Center Ethics Committee (numbered J2022-79-2022-1-3).

### Patients

Among cancer patients admitted to the Shizuoka Cancer Center, who were referred to the palliative care team (PCT) for symptom management between January 2016 and November 2022, we included cases in which opioids had not been administered prior to the start of the PCT intervention and the introduction of opioids with injectable drugs was required for cancer pain.

### Study design

All the patients, who had never received any opioid and administered combination therapy with OPI and tramadol by the PCT as the initial opioids for their cancer pain from January 2016 to November 2022, were collected and reviewed. The following measurements were investigated retrospectively with the electronic medical chart: patient demographics, primary cancer sites, mechanism of clinically diagnosed pain, types of OPI injection, OPI and tramadol doses, pain intensity, numbers of rescue medication, adverse events, concomitant medications, and outcomes were investigated. The clinical classification of pain was decided by the head author, who is a certified pain clinician with more than 25 years of clinical experience in the PCT, according to physical findings and image studies. No specific scale(s) was used to differentiate pain types. Outcome measures were performed until the patient was discharged, eventually until he/she died (for 1–35 days).

### Protocol of combined OPI + tramadol injection administration

OPI and tramadol injections were combined, diluted with physiological saline as needed for a total volume of 10 mL, and continuous intravenous or subcutaneous administration was performed. Table 1 shows examples of mixing OPI and tramadol injection doses. The initial doses of OPI and tramadol injections were set by the PCT members. Rescue medication was provided with a bolus of 1- or 2-hour dose, and the repeated use was possible when the interval was longer than 30 minutes. The dose was adjusted to make the intensity of pain 4/10 or less in Numerical Rating Scale (NRS) or 1 or less in Support Team Assessment Schedule-Japanese (STAS-J).^16^ The PCT examined the patients face-to-face every day to see if the dose would need to be increased or decreased, but the ward nurses were also empowered to adjust the dose within the range of instructions set by the PCT physicians (Table 2). After the patient was transferred to the palliative care unit (PCU), the dose was adjusted by the physician in chief, the palliative care physicians, and the PCU nurses.

**Table 1.** Examples of OPI + Tramadol Mixture

μ-opioid	Conc. (%)	Dose (mL)			Infusion rate (mL/h)	Daily dose (mg)	
		μ-opioid	Tramadol	Saline		μ-opioid	Tramadol
Morphine	1	1	2	7	0.1	2.4	24
		2	4	4		4.8	48
		5	5			12	60
	4	5	5			48	60
Oxycodone	1	1	2	7		2.4	24
		5	5			12	60
Hydromorphone	0.2	5	5			2.4	60
	1	2	4	4		4.8	48

※ Intravenous or subcutaneous tramadol administration is off-label in Japan.

**Table 2.** Protocol of OPI + Tramadol Formulation and Dose Adjustment

• Hydromorphone low concentration injection (2 mg/1 mL) 1A + tramadol injection (100 mg/2 mL) 1A + saline 7 mL (total 10 mL)
0.2 mL/h (0.96 mg/day of hydromorphone, 48 mg/day of tramadol)
** **Continuous IV or continuous subcutaneous injection
• Rescue medication:
Hydromorphone + tramadol injection combination fast-forwarding with 1- to 2-hour volume
(It can be repeated with an interval of at least 30 minutes)
• If more than three rescues are required in each 8-hour work period:
The rate of hydromorphone + tramadol injection combination can be increased with increments of 0.05 mL/h (up to 0.4 mL/h).
• In case of severe somnolence or apnea for more than 10 seconds:
The rate of hydromorphone + tramadol injection combination may be tapered with increments of 0.05 mL/h (down to 0.1 mL/h).

### Measurements

Age, gender, performance status eastern cooperative oncology group (ECOG), albumin-bilirubin grade (ALBI) score as hepatic reserve, estimated glomerular filtration ratio (eGFR) as renal function, concurrence of dyspnea, necessity of palliative sedation, duration of OPI and tramadol treatment, and patients’ outcome were recorded. Pain intensity was assessed routinely at least three times a day (once during each 8-hour work shift) as average value, and before each rescue medication as worst one, and 30–60 minutes after the rescue to confirm its efficacy. Pain intensity was assessed by PCT members and/or ward nurses during patient stay in a general ward. After a patient was transferred to the PCU, it was assessed by a palliative care physician and/or PCU nurses.

Mean, standard deviation (SD), and median of the measurements were calculated by an analysis program in Microsoft Excel software.

### Statistical analysis

Average pain intensity in NRS and STAS-J scores were statistically analyzed using Wilcoxon signed-rank sum test. A *p* value <0.05 was considered statistically significant.

## Results

### Patient demographics

Of the 632 inpatients who had been referred to the PCT within the study period, 116 were opioid-naïve patients who had never been administered any opioid prior to the treatment by our PCT (Table 3). A total of 44 patients who were treated with opioids using OPI + tramadol injections for pain relief were found and eligible for this study.

**Table 3.** Demographic Data of the Patients

Age (years)	74.0 ± 9.8^*^ (range: 47∼90, median: 67)				
Gender (*n*)	Male:female = 24:20				

PS (EGOG) (*n*)	0	1	2	3	4
	1	9	12	12	10

ALBI score (*n*)	1	2a	2b	3
	0	3	13	28

eGFR	63.1 ± 35.4^*^ mL/min/1.73 m^2^				
Dyspnea (*n*)	Yes:no = 6:38				
Sedation (*n*)	Yes:no = 14:30				
Period (days)	12.0 ± 8.2^*^ (range: 1*–*35 median: 16)				
Outcome (*n*)	Died in general wards : died in PCU = 28:16				

^*^ Mean ± SD.

eGFR, estimated glomerular filtration rate; PCU, palliative care unit; PS, performance status; SD, standard deviation.

Their ages ranged from 47 to 90 years (mean 64.1 years, median 67 years) and males accounted for 24/44.

**FIG. 1.**
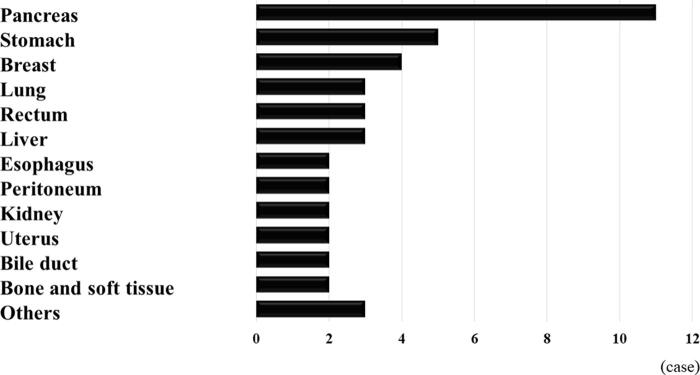
Organs of primary cancer.

The organs of primary cancer were pancreas in 11 patients, stomach in 5, breast in 4, lung, rectum, and liver in 3, esophagus, peritoneum, kidney, uterus, bile duct, and bone and soft tissue in 2 for each, bladder, myelodysplastic syndrome, and unknown in 1 for each (Fig. 1).

**FIG. 2.**
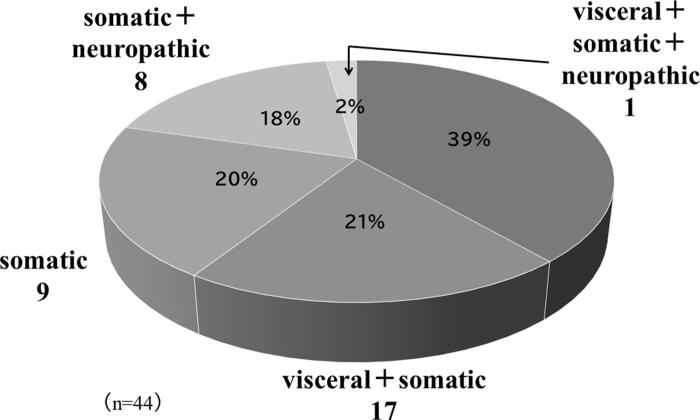
Clinical classification of causes of cancer pain.

Clinical classification of pain included visceral pain + somatic pain in 17 patients, visceral pain in 9, somatic pain in 9, somatic pain + neuropathic pain in 8, and visceral pain + somatic pain + neuropathic pain in 1 patient (Fig. 2).

The performance statuses (ECOG) scoring 0, 1, 2, 3, and 4 indicated in 1, 9, 12, 12, and 10 cases, respectively. ALBI scores, the indicators of liver function reserve, were graded as 1, 2a, 2b, and 3 in 0, 3, 13, and 28 cases, respectively. The eGFR was 63.1 ± 35.4 (mean ± SD) mL/min/1.73 m^2^. Dyspnea accompanied in 6 cases.

In addition, the continuous palliative sedation was required for severe insomnia and delirium, etc., in terminal stage in 14 cases.

One patient with gastric cancer received chemotherapy during the study period. No patients received radiotherapy. In addition, neither nerve block nor interventional radiological technique was performed to treat pain for these 44 patients.

### Details of treatment

**FIG. 4.**
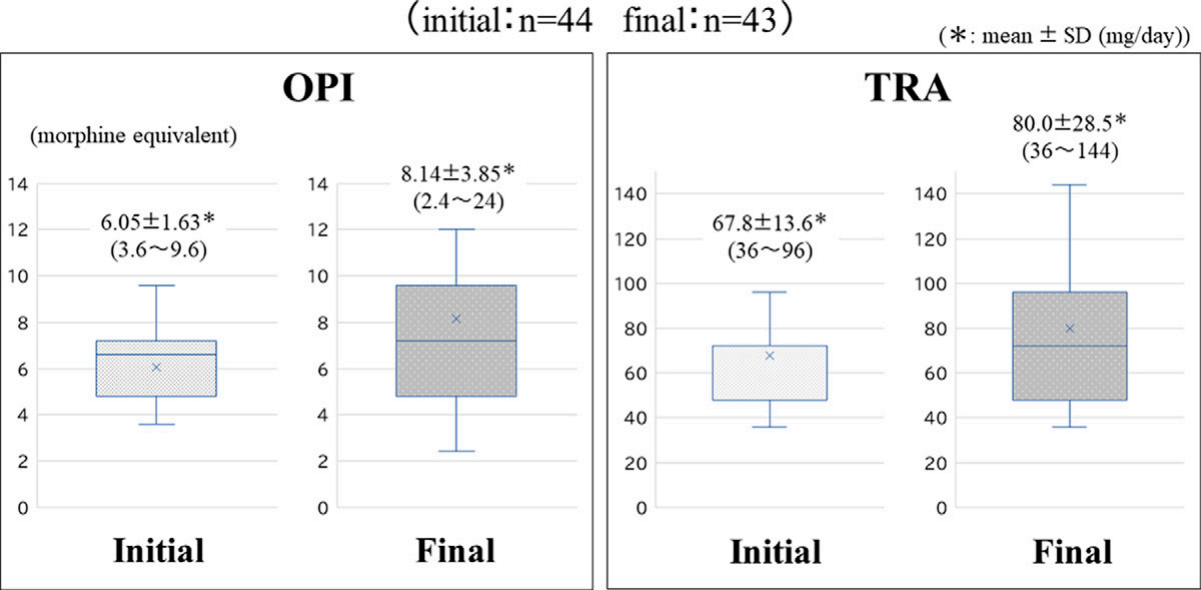
Doses of OPI (left) and TRA (right) during the initial 24 hours and the final 24 hours. OPI, pure-μ-opioid receptor agonist.

The duration of treatment with the PCT or in the PCU was 12.0 ± 8.2 days (mean ± SD) (1–35 days, median 16 days) (Table 4 and Fig. 4). Sixteen of the patients moved from the general wards to the PCU during the treatment.

**Table 4.** Doses of OPI and Tramadol During the Initial 24 Hours (Left) and the Final 24 Hours (Right), Opioid Switching, and the Frequency of Rescue Doses During the Study Period

	Initial 24 hours	Final 24 hours
OPI injection*	6.05 ± 1.63^**^ mg/day (3.6*–*9.6 mg/day) Median: 4.8 mg/day	8.14 ± 3.85^**^ mg/day (2.4*–*24 mg/day) Median: 6.0 mg/day
Tramadol injection	67.8 ± 13.6^**^ mg/day (36*–*96 mg/day) Median: 48 mg/day	80.0 ± 28.5^**^ mg/day (36*–*144 mg/day) Median: 72 mg/day
OPI switching (*n*)	Hydromorphone → morphine: 1 Morphine → hydromorphone: 1 Hydromorphone → tapentadol: 1	
Number of rescue medication	0.9 ± 1.5^**^ times/day (0*–*8 times/day) Median: 0 times/day	1.1 ± 1.8^**^ times/day (0*–*8 times/day) Median: 0 times/day

^*^ Oral morphine equivalent.

^**^ Mean ± SD.

OPI, pure-μ-opioid receptor agonist.

**FIG. 3.**
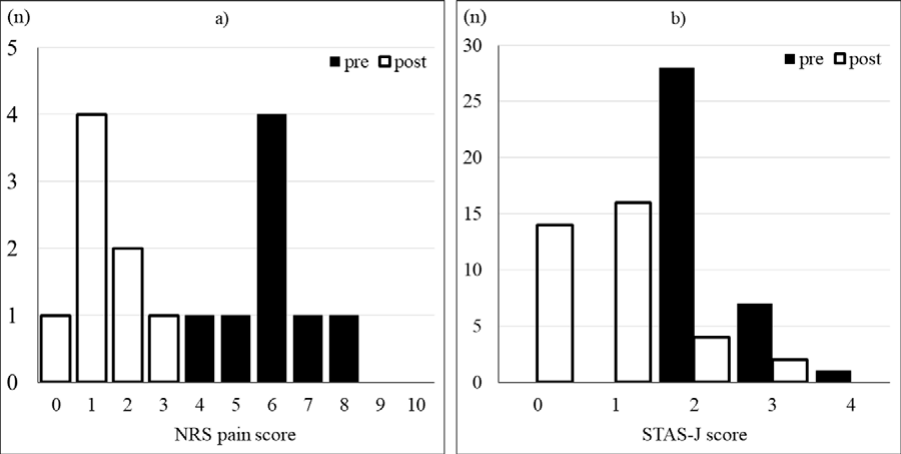
Changes in NRS pain score **(a)** and STAS-J score **(b)**. White and black bars indicate the average pain intensity on the day before and the day after the start of the treatment, respectively. Both NRS and STAS-J scores significantly diminished after the treatment (*p* < 0.05). NRS, Numerical Rating Scale; STAS-J, Support Team Assessment Schedule-Japanese.

The types of OPI injections (Fig. 3) administered were hydromorphone in 38 patients, morphine in 3, oxycodone in 2, and fentanyl in 1 at the beginning of the treatment and hydromorphone in 38 patients, morphine in 2, oxycodone in 2, and fentanyl in 1 toward the end of the treatment (death). Hydromorphone was selected in majority of the patients because it can be relatively safely administered for patients with renal dysfunction. During the treatment, one patient switched from hydromorphone to morphine owing to dyspnea, one patient switched from morphine to hydromorphone owing to intolerable sleepiness, and one patient switched from morphine plus tramadol to tapentadol sustained-release tablet because he became able to take medications orally.

The doses of OPI injections were 6.05 ± 1.63 mg/day (mean ± SD) (3.6–9.6 mg/day, median 4.8 mg/day) at the beginning of the treatment and 8.14 ± 3.85 mg/day (mean ± SD) (2.4–24 mg/day, median 6.0 mg/day) toward the end of the treatment. The doses of tramadol injections were 67.8 ± 13.6 mg/day (mean ± SD) (36–96 mg/day, median 48 mg/day) at the start of the treatment and 80.0 ± 28.5 mg/day (mean ± SD) (36–144 mg/day, median 72 mg/day) toward the end of the treatment (Table 4).

### Concomitant medications

The concomitant medications administered before the OPI + tramadol combination therapy were acetaminophen in 22 patients, loxoprofen Na in 3, diclofenac in 1, celecoxib in 1, flurbiprofen axetil in 1, dexamethasone in 1, betamethasone in 1, prednisolone in 1, methylprednisolone in 1, medroxyprogesterone acetate ester in 1, and pregabalin in 3.

In addition, following drugs were administered during the OPI + tramadol combination therapy; acetaminophen in 3 patients, diclofenac Na transdermal patch in 1, betamethasone in 14, water-soluble prednisolone in 2, methylprednisolone in 1, ketamine in 1, and lidocaine systemic injection in 2. These medications were administered as coanalgesics/adjuvants and/or to relieve symptoms other than pain such as pyrexia and cancer-related fatigue.

In 26 patients who did not have these concomitant medication(s), the doses of OPI injections were 6.14 ± 1.99 mg/day (mean ± SD) (3.6–9.6 mg/day, median 4.8 mg/day) at the beginning of the treatment and 6.37 ± 2.65 mg/day (mean ± SD) (2.4–12 mg/day, median 4.8 mg/day) toward the end of the treatment. The doses of tramadol injections were 69.8 ± 17.9 mg/day (mean ± SD) (48–96 mg/day, median 72 mg/day) at the start of the treatment and 74.2 ± 35.8 mg/day (mean ± SD) (48–120 mg/day, median 72 mg/day) toward the end of the treatment.

### Analgesic effects

The average daily pain intensity (ADPI) on the day before starting the OPI and tramadol combination therapy was assessed as 4, 5, 6, 7, and 8 on NRS (0–10) in 1, 1, 4, 1, and 1 in 8 patients (who were able to assess NRS pain scale), respectively. In the remaining 36 patients who were unable to use NRS pain scale, pain intensity was assessed using STAS-J score. Their STAS-J scores were 2, 3, and 4 in 28, 7, and 1 patients (pain intensity in these 36 patients was difficult to be scored in NRS), respectively. ADPI on the next day (within 24 hours) of starting the therapy diminished to 0, 1, 2, and 3 on NRS in 1, 4, 2, and 1 in 8 patients, respectively. In the remaining 36 patients, STAS-J scores also diminished to 0 and 1 in 14 and 16 patients, respectively, within 24 hours after the initiation of the therapy, however, STAS-J scores had remained as 2 and 3 in 4 and 2 patients, respectively. The change in both ADPI in NRS and STAS-J score (Fig. 3) were statistically significant (*p* < 0.05).

The numbers of rescue doses for pain on the day before starting the therapy and during the final 24 hours of the treatment were 0.9 ± 1.5 (range; 0–8, median: 0) times/day and 1.1 ± 1.8 (range: 0–8, median: 0) times/day, respectively (Table 4).

### Adverse events

No serious adverse events other than well-known side effects of OPI or tramadol injection, such as mild-to-moderate sleepiness (mild in 13, moderate in 1) and mild nausea (in 2) in the early stages of the initiation, were observed. No apparent serotonin syndrome-like symptoms such as spasticity, psychiatric symptoms, tremor, or tachycardia were observed either. There were no abnormal reactions of veins or subcutaneous tissues at the injection sites.

### Patient outcomes

In total, 28 patients died as-is after continuing hospitalization in the general wards and 16 died after having been moved to the palliative care ward owing to cancer progression (i.e., after the transition of intervention from the PCT to the PCU staff with the physicians for palliative medicine acting as their main physicians) (Table 3). No patients were able to be discharged. And, there were no patients who were able to stay out overnight during the treatment either.

## Discussion

### OPI and tramadol in cancer pain treatment

Pain is a frequent symptom for cancer patients and considered to be the greatest fear for the patients.^1,2^ Pharmacotherapy is the mainstay of cancer pain treatment,^3,4^ and OPI still plays a vital role for relieving moderate-to-severe pains. OPI exerts analgesic effects in a dose-dependent manner, but with the increase in OPI, side effects in the central nervous system such as drowsiness and cognitive decline, gastrointestinal side effects such as nausea and vomiting, and overdose administration are prone to reduce QOL of the patients and the care-givers, alongside of cancer-related fatigue and anorexia.

Therefore, if adverse events can be minimized while maximizing the analgesic action of OPI to obtain higher-quality analgesia, it will contribute significantly to the improvement of the patients’ QOL. Nonopioid analgesics, adjuvants, and nonpharmacological approaches such as nerve blocks, radiotherapy, and interventional radiology can also be used complementarily for OPI analgesia to improve the QOL of the patients. However, it is often the case that these treatments are not indicated depending on the patient’s condition, and pharmacotherapy remains as the main analgesic method for the patients, as it can be provided any time.

Therefore, taking advantage of the characteristics of tramadol described below, we have tried to combine OPI with dual-action analgesics such as tramadol to achieve higher-quality analgesia. In addition, recent reports have indicated that interventions increasing the levels of serotonin in the hippocampus might counteract cognitive impairment caused by opiates,^17^ which reduce hippocampal neurogenesis.^18^ Tramadol may eventually improve morphine-induced cognitive dysfunction by increasing neurogenesis in the hippocampus.

### Significance, efficacy, and safety of the combination of OPI and tramadol

Tramadol is one of the dual-action analgesics with two optical isomers suppressing serotonin and noradrenaline reuptake at the dorsal horn of spinal cord, and the active metabolite, o-desmethyltramadol, acts as a μ-opioid receptor agonist.^6,11^ In addition, animal experiments^13,14^ and a clinical report on postoperative analgesia^15^ have shown that tramadol synergizes the analgesic action in combination with morphine, although this combination does not show any adverse effects in either additive or synergistic manner. Therefore, the combination of OPI and tramadol may provide sufficient analgesic activity along with reducing OPI dose, resulting in minimum side effects of opioids.

This is considered as a desirable method in terms of efficacy and safety as a treatment for pain in advanced and terminal cancer patients, who are often frail and susceptible to adverse effects. Focusing on these points, the authors have innovated an opioid therapy to treat cancer pain with this concomitant injection of OPI and tramadol since 2016, and as part of our clinical experiences, we conducted this study to elucidate safety and efficacy of this method. Although the patients who were eligible for this study had relatively short-term prognoses, they were able to obtain sufficient analgesia with OPI and tramadol as opioid-induction drugs, and there were no adverse events other than already-known side effects such as drowsiness that could be caused by OPI or tramadol injection. In addition, although the number of study cases was small, we had impressions that the time from the start of administration to obtaining sufficient analgesia was relatively short because pain in all the patients were relieved within 24 hours and that the increase rate of the OPI infusion dose was small.

Although intramuscular administration is the only indication for tramadol injection in Japan, the safety and efficacy of tramadol injection have been established, and there is no significant difference in analgesic effect between intravenous and subcutaneous administrations.^19,20^ In fact, we were able to administer tramadol injection without any problem in this study, including adverse reactions at the injection sites.

Moreover, tramadol is also effective for neuropathic pain and its administration is recommended in the several guidelines for neuropathic pain treatment.^21,22^ Patients who were unable to take oral medication were included in this study, and although it was impossible to administer analgesic adjuvants such as anticonvulsants and antidotes orally, sufficient improvement of symptoms could be obtained even in the patients with neuropathic pain (approximately 20% of the patients included in this study). Our results also suggest that the addition of tramadol may have greater efficacy for neuropathic pain than OPI alone. It is noteworthy that rescue medications were not required frequently during the period from the initiation of administration to the end of it, and although it was relatively short, the fact that much increase in OPI injection was not required suggests that synergistic analgesic effects may have been obtained by combining tramadol with OPI. However, in order to prove this, prospective comparative studies with OPI injection alone in patients with similar backgrounds will be needed.

Serotonin syndrome is a serious side effect of tramadol that is not observed in OPI,^9,23–25^ but there were no symptoms suspected of serotonin syndrome in this study. This seems to be the result of setting the starting dose to be small and adjusting the dose along with watching the renal function. Since metabolic excretory function is often impaired in patients with terminal stage cancer, even more careful dose setting may be required, especially in first-time cases.

### Limitations

Since this study was performed in a retrospective design with a limited number of patients and the combination of nonopioid analgesics and analgesic adjuvants differed for each patient, it cannot be ruled out that analgesic effects induced by drugs other than OPI + tramadol infusion may have also influenced the outcome. Furthermore, we could not suggest superiority of this innovative combination therapy to a conventional opioid monotherapy in efficacy, for example, less opioid escalation index or preferable profile in adverse effects. If the intent is to look at efficacy, a randomized controlled trial is required, and if the aim is to understand the adverse effects of OPI + tramadol combination therapy, a prospective pharmacovigilance study of routine practice in a real-world setting, with an assessment of casualty for more severe adverse drug reactions, would be the optimal design. Therefore, prospective multicenter pharmacovigilance studies are necessary to elucidate clinically relevant advantages and precise adverse events of this combination therapy and to generalize it as one of the standard opioid therapies to relieve cancer pain in the future.

In addition, the current study has multiple methodological issues: a lack of standardized assessments of pain and adverse drug reactions, confounding with other medications, and adverse drug reactions are never well recorded or assessed outside of a study. As there are many cases where the quantification of pain by NRS was difficult, and it was uncertain whether the patients and their families were satisfied with the treatment, we assessed pain using STAS-J. Because the patients were approaching the end-of-life stage and going through rapid changes in the conditions, it was often difficult to objectively evaluate their pain intensity with the numerical values fluctuating greatly depending on the timing of evaluation. Therefore, there were many cases where we had to rely on other pain intensity scales, such as STAS-J, than NRS.

In view of this point, we conducted the adjustment of the dosage of the drugs as immediately as possible based on the subjective evaluation by the patients and their families as well as the objective evaluation by the PCT with multiprofessional types, the general ward staff, and the PCU staff.

Regarding adverse events, such as drowsiness and nausea, they could have been caused by OPI + tramadol infusion, but also have been owing to their primary diseases. As the study subjects were the cancer patients with short prognoses, it was sometimes difficult to accurately differentiate the symptoms with the primary diseases from the complications or the side effects of the drugs.

Initiation of the treatment was performed by the PCT members, but in 36% of the patients, the treatment was passed on to the palliative care ward staff in the middle. However, because all the physicians in the palliative care wards belong to the Division of Palliative Medicine and have clinical experiences in the PCT, it is unlikely that they would make significant changes in terms of the pain assessment and the dose adjustment of OPI and tramadol.

### Future perspectives

From the methodological point of view, prospective studies that measure pain intensity and adverse effects using a validated tool will be required after further standardization of the dosage and dose adjustment of concomitant OPI + tramadol infusion.

Clinically, there are some serious problems in this treatment including the facts that intravenous or subcutaneous administration of tramadol injection is not indicated for public health insurance coverage, and that tramadol injection cannot be used in home care settings in Japan. In addition, optimal formulation ratio of OPI and tramadol, optimal rescue dosing, and the safety of it remain as issues to be clarified. Also, how to prepare and provide optimal oral OPI and/or dual-action analgesics such as tramadol and tapentadol when the patient can take oral medications will need to be formulated. Further examination will be necessary based on the above-mentioned issues in the future.

Finally, we are considering to investigate and show the efficacy and safety of this combination therapy with OPI and tramadol injections for opioid-tolerant patients with cancer pain in the next step. Furthermore, we are planning to perform a prospective randomized controlled trial to compare this method with standard opioid monotherapy.

## Conclusion

Our findings in this clinical analysis suggest that our innovative and unique combination therapy using OPI + tramadol injection is a safe and effective method to introduce opioid for opioid-naïve patients with cancer pain.

## Abbreviations Used

ALBI score: Albumin-Bilirubin Grade score
DAA: dual-action analgesic
ECOG: Eastern Cooperative Oncology Group
eGFR: estimated glomerular filtration rate
NRS: numerical rating scale
OPI: μ-opioid receptor agonist
PCT: palliative care team
PCU: palliative care unit
QOL: quality of life
SD: standard deviation
STAS-J: Support Team Assessment Schedule-Japanese
